# More accurate recombination prediction in HIV-1 using a robust decoding algorithm for HMMs

**DOI:** 10.1186/1471-2105-12-168

**Published:** 2011-05-17

**Authors:** Jakub Truszkowski, Daniel G Brown

**Affiliations:** 1 David R Cheriton School of Computer Science, University of Waterloo, Waterloo ON N2L 3G1 Canada

## Abstract

**Background:**

Identifying recombinations in HIV is important for studying the epidemiology of the virus and aids in the design of potential vaccines and treatments. The previous widely-used tool for this task uses the Viterbi algorithm in a hidden Markov model to model recombinant sequences.

**Results:**

We apply a new decoding algorithm for this HMM that improves prediction accuracy. Exactly locating breakpoints is usually impossible, since different subtypes are highly conserved in some sequence regions. Our algorithm identifies these sites up to a certain error tolerance. Our new algorithm is more accurate in predicting the location of recombination breakpoints. Our implementation of the algorithm is available at http://www.cs.uwaterloo.ca/~jmtruszk/jphmm_balls.tar.gz.

**Conclusions:**

By explicitly accounting for uncertainty in breakpoint positions, our algorithm offers more reliable predictions of recombination breakpoints in HIV-1. We also document a new domain of use for our new decoding approach in HMMs.

## Background

We consider the problem of locating recombination breakpoints in viral genomes. The Human Immunodeficiency Virus has been classified into several major phylogenetic groups called *subtypes *[1]. Recombination between those groups is common [2], so a viral genome can be composed of several regions arising from different subtypes. Given a viral genome sequence, we would like to identify whether it is a recombinant, and which subtypes gave rise to which regions of its sequence.

Identifying recombinations is important for epidemiological monitoring of the virus, as well as for the design of potential vaccines and treatments. For example, some subtypes develop more resistance to anti-retroviral drugs than others [3]. Accurate identification of recombination patterns is also crucial to detecting superinfections in patients [4].

**Figure 1:**
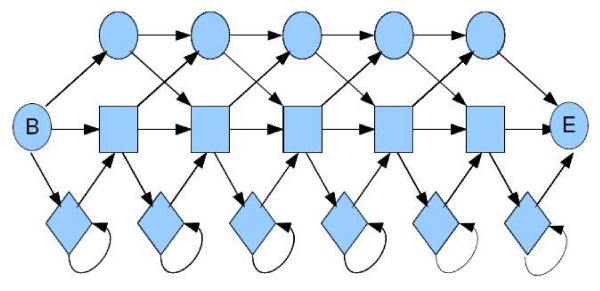
**A profile hidden Markov model**. A profile HMM for an alignment with 5 consensus columns. Rectangles represent match states. Diamonds represent insert states. Circles represent delete states as well as (silent) begin and end states.

**Figure 2:**
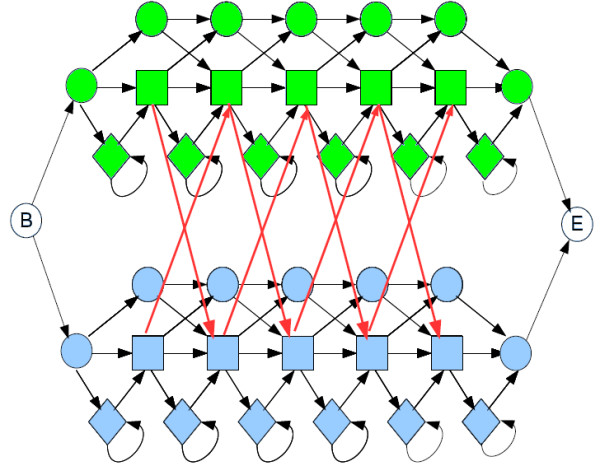
**A jumping profile hidden Markov model**. A jumping profile hidden Markov model for 2 subtypes. States sharing the same label are coloured with the same colour. Red arrows indicate jump transitions between subtypes. For clarity, jumps involving insert or delete states have been omitted.

One current method for detecting recombinations uses profile HMMs; we describe others in the next section. Profile HMMs are a widely used tool in modelling families of sequences and can be thought of as a way of summarizing multiple sequence alignments of a sequence family [5]. A profile HMM is composed of three sequences of *match*, *insert *and *delete *states. Match states correspond to consensus columns in the multiple alignment. Each match state can make a transition to a corresponding insert state, which models residues appearing between consensus columns. Finally, a chain of silent *delete *states accounts for the possibility of a sequence skipping some consensus columns in the alignment. Figure 1 shows an example of a profile HMM. Profile HMMs are derived from multiple sequence alignments using standard algorithms [6]. The *jumping profile hidden Markov model *(jpHMM) [7], as shown in Figure 2, represents each HIV subtype as a profile HMM derived from an alignment of sequences from this subtype. Transitions between each pair of subtypes are added in each column to model possible recombinations between subtypes. These transitions are called *jumps *and are assigned very low probability, as the number of recombinations in a sequence is low compared to its total length. Predicting recombinations is achieved using the classical Viterbi algorithm (see *e.g*. [6]). Schultz *et al*. [7,8] show that using the jpHMM with the Viterbi algorithm to predict recombinations outperforms Simplot, then the most commonly used recombination detection tool.

We apply a novel algorithm to further improve the accuracy of predictions provided by a jpHMM. Our key observation is that in many cases, high similarity between different subtypes makes it impossible to pinpoint breakpoint positions. We focus on approximately predicting breakpoint positions rather than maximizing the probability of being exactly correct. We have demonstrated the efficiency of a similar decoding strategy for transmembrane protein topology prediction in previous work [9], where we also developed much of our algorithmic framework.

### Related work

Many tools have been developed for HIV recombination detection. These tools can be divided into two groups: those based solely on comparisons between sequences and those which are additionally based on phylogenetic reconstruction.

Among the tools based solely on sequence comparison, Simplot [10] is perhaps the most well-known. It provides a graph showing the local similarity of a query sequence to a set of reference sequences. Another tool, RIP [11] also operates on the same principle. The *jumping profile hidden Markov model *(jpHMM) [7] uses profile hidden Markov models to model different subtypes and predict recombinations. Here, comparisons are made between the query sequence and profile HMMs describing *collections *of sequences belonging to the same subtype.

Tools using phylogenetic information try to detect recombinations by reconstructing phylogenies for different regions of sequence alignment and comparing them to detect topology changes, which suggest recombination took place. The REGA HIV subtyping tool [12] uses a sliding window approach to reconstruct phylogenies for different regions in the alignment, though it only identifies the subtypes without predicting breakpoint positions. DualBrothers [13] and cBrother [14] divide the sequence alignment into segments with different evolutionary parameters and different phylogenetic histories. They then use Markov Chain Monte Carlo to sample from the posterior distribution of such partitions and their corresponding trees. GARD [15] uses a genetic algorithm to find a maximum-likelihood partition of the alignment into segments with different evolutionary histories. De Oliveira Martins and Kishino [16] try to account for uncertainty of segment assignments by using a distance measure between mosaic structures and applying a centroid estimator.

Several phylogeny-based tools use *phylogenetic HMMs *(phylo-HMMs) to model topology changes along the sequence alignment. Each state in a Phylo-HMM corresponds to a phylogenetic tree and emits a column of the multiple sequence alignment. Husmeier and Wright [17] use a phylogenetic HMM with three states to detect recombinations between four sequences. Kedzierska and Husmeier [18] propose a heuristic technique using an HMM that emits tree topologies. Westesson and Holmes [19] use a version of the EM algorithm to train a phylogenetic HMM with a fixed, user-defined number of tree topologies. Webb, Holmes and Hancock [20] integrate out the branch lengths in the phylogenies to speed up the MCMC inference.

We note that the HMMs used in these phylogeny-based approaches are very different from the jpHMM model we employ. A state in a phylo-HMM emits a *column *in the multiple sequence alignment of the analyzed sequences. In contrast, a jpHMM is a single-sequence HMM where each state emits a base of the sequence under analysis. While phylogeny-based methods take a multiple sequence alignment as an input, the jpHMM's input is only the single recombinant sequence to be analyzed: the multiple alignments already built are encapsulated in the probabilistic properties of the profile HMM. A state path through the jpHMM corresponds to an assignment of regions of the input sequence to the subtypes modelled by the profile HMMs.

Phylogeny-based methods provide more flexibility in modelling sequence evolution, but they often require the knowledge of evolutionary parameters to work properly and are computationally expensive. In contrast, profile-based approaches are simpler. They do require parameters that summarize existing multiple alignments, though this is perhaps easier data to infer than are evolutionary rates. Profile methods are also typically faster, as they do not require the evaluation of tree likelihoods for a number of different topologies; their computation is also accelerated using heuristics such as beam search [21]. Phylogenetic approaches which do not enumerate over many tree topologies, but instead efficiently compute the optimal ones, could in principle give fast runtimes, but have not been used widely for HIV.

The jpHMM does not use any phylogenetic information, but it differs from other phylogeny-free approaches in that it compares the input sequence to profile HMMs rather than single sequences representing different subtypes. Here, we focus on improving the prediction accuracy of jpHMM by applying a new decoding algorithm to infer recombination breakpoints.

Recently, some other approaches have also been proposed to account for uncertainty of boundary locations in recombination detection. Schultz *et al*. [8] detect *uncertainty regions *in the Viterbi prediction where the posterior probability of the predicted subtype is below a certain threshold. Nánási *et al*. [22] devised a novel decoding algorithm for HMMs which maximizes the expected number of breakpoints predicted up to a certain error tolerance, but does not assess the joint probability of all breakpoints; more detail is in Nánási's thesis [23].

These methods have drawbacks. The algorithm by Nánási *et al*. is too computationally expensive to be practical on full-length viral sequences and has only been evaluated on a small region of HIV alignment. The newer approach of Schultz *et al*. [8] still uses the Viterbi algorithm to locate breakpoints, although it gives more insight into their credibility.

While our approach bears some resemblance to the two methods mentioned above, it differs from both in that it optimizes a global objective while allowing for small local errors in our prediction.

### Our approach

Schultz *et al*. [7] use the classical Viterbi algorithm for jpHMM decoding, despite its many drawbacks. The Viterbi path may have extremely small probability and is unlikely to be correct overall for sequences having more than a few elements. Also, we are interested in finding a correct *labelling*, rather than an optimal state path in the model. In many situations, likely labellings consist of a large number of low-probability paths. In such cases, the Viterbi algorithm chooses the labelling that corresponds to the most likely state path, but the posterior probability of such labelling may be much lower than the posterior probability of a labelling composed of many low-probability paths. This phenomenon is known as the *path/labelling problem *[24]. Even in the absence of the path/labelling problem, the Viterbi path is unlikely to be exactly correct in cases where there is much uncertainty about exact border positions. Many different labellings will then have similar probabilities, and the most probable labelling will not necessarily be correct.

In our previous work on robust HMM decoding [9], we ameliorate some of these problems by searching for the centre of the most probable *ball *of predictions rather than a single prediction. A ball of radius *r *centred at a specific labelling *λ *for the sequence contains a number of paths with labellings at distance less than an upper bound *r *from *λ*. The number of such paths grows exponentially with *r*. We often find balls of large posterior probability, giving us more confidence that the centre of the ball is close to the true labelling. We define balls on labellings rather than paths, so the path/labelling problem is avoided.

Our ball algorithms, with our border shift distance (defined in the next section), are a natural choice for the problem of recombination prediction. Our priority in this problem is accurately predicting which subtypes have occured in our sequence. Since subtypes are highly similar in many places, exactly predicting breakpoints appears impossible, meaning that we should instead focus on approximately predicting breakpoints. Where subtype similarity is low, we obtain quite accurate predictions by shrinking the ball radius.

### Preliminaries

A labelled HMM is defined by a transition matrix *A*, an emission matrix *E*, and a labelling function *l*. The matrix *A *is a stochastic *m *× *m *transition matrix where *a_ij _*gives the probability of transition from state *i *to state *j*; *E *is an *m *× |∑| emission matrix where *e_kσ _*gives the probability of emitting symbol *σ *∈ ∑ in state *k*. The labelling function *l *assigns to each state a single label from the set Λ of possible labels. Many states often share a single label. We assume that the model starts in a state *x*_0_.

In the context of recombination detection with jpHMM, each state corresponds to a column in the alignment of sequences from a particular subtype. For each subtype and each consensus column in the alignment of the subtype, we have a match, insert, and a delete state. Each state is labelled by the subtype it belongs to. An HMM constructed this way has a large number of states: around 3 · 9000 · 14 = 378000, where the factor of 9000 is the number of consensus columns in each subtype alignment, and 14 is the number of subtypes modelled by the jpHMM. This number of subtypes is higher than the usual number of HIV subtypes reported in the literature [1] because the creators of the jpHMM-HIV chose to model the sub-subtypes A1, A2, F1 and F2, and the CRF 01_*AE*, as well as HIV group O and the Simian Immunodefficiency Virus, as separate subtypes [7]. The number of labels is 14, one for each subtype.

A *path *in an HMM is a sequence of states *x *= *x*_0_, *x*_1_, . . . , *x_n _*visited by the model at each step. We denote the corresponding sequence of emissions as *y*_1_, . . . , *y_n_*. The *labelling λ *corresponding to path *x *is defined as *λ *= *ℓ*(*x*_0_), *ℓ*(*x*_1_), *ℓ*(*x*_2_), . . . , *ℓ*(*x_n_*). For a generic sequence *z*_1_, . . . *z_n_*, we write to denote the subsequence *z_i_*, . . . , *z_j_*. We write  to denote *z_i_*, . . . , *z_j _*= *u*, . . . , *u*.

Since a typical sequence contains only a few breakpoints, a labelling for a sequence will usually have many consecutive positions with the same label. Given a labelling *λ *= *ℓ*(*x*_0_), *f*_1_, *f*_1_, . . . , *f*_1_, *f*_2_, *f*_2_, . . . , *f*_2_, . . . , *f_k_*, . . . , *f_k_*, which consists of *λ*_0 _followed by a number of positions labelled *f*_1_, then a number of positions labelled *f*_2 _(which is different from *f*_1_), and so on, we define its *footprint *to be the sequence *f *= *f*_1_, . . . , *f_k_*; this corresponds to the overall labelling of the sequence, but with the label boundaries entirely flexible. Typically, regions with the same label correspond to features: in our application, they are sequence intervals derived from a single subtype. Footprints are typically much shorter than a sequence of emissions.

Consider two different labellings *λ *and *λ*' for the same sequence *y*. We define their *border shift distance d*(λ, λ') as a function of the feature boundaries, as follows.

**Figure 3:**
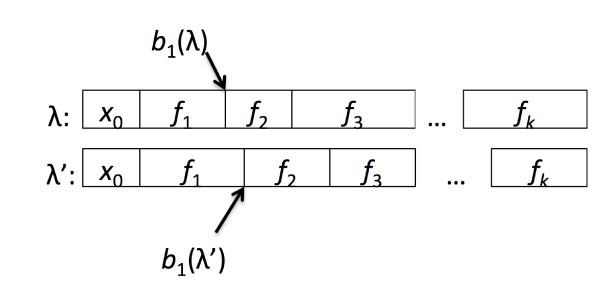
**The position of borders in labellings**. The *i*th border for a labelling *λ*, *b_i_*(*λ*), is the first position in *λ *with the label *f*_*i*+1_. The shift in the *i*th border, between two labellings *λ *and *λ' *that share the same footprint *f *is |*b_i_*(λ) - *b_i_*(*λ'*)|.

First, if *λ *and *λ*' have different footprints, they are incompatible; their distance is ∞. Otherwise, let *f *= *f*_1_, . . . , *f_k _*be their common footprint, and let *b_i_*(*λ*) be the position in *λ *corresponding to the first emission from the interval labelled *f*_*i*+1_. The border shift distance between *λ *and *λ*' is then max_*i *= 1 ... *k*-1 _|*b_i_*(λ)-*b_i_*(λ')| it is the maximum boundary shift between the corresponding features in two labellings. Figure 3 shows how we compute this distance.

A *ball *of radius *r *centered at a labelling *λ*, denoted as *B*(*λ*, *r*), is defined as the set of all labellings *λ*' having distance at most *r *from *λ*.

## Methods

### Computing ball probabilities

Our first algorithm computes, for a given labelling *λ*, the probability of a ball of radius *r *centered at *λ*. We use a variant of the traditional forward algorithm for HMMs [6], which computes the probability that an HMM generates a sequence *y*, in *O*(*nm*^2^) time.

**Figure 4:**
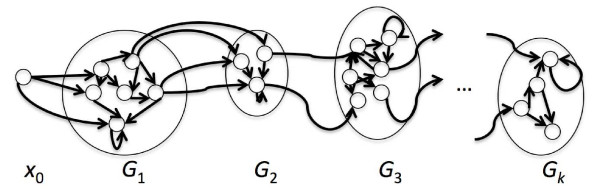
**Computing the probability of a footprint**. To compute the probability of a footprint *f *= *f*_1_, . . . , *f_k_*, we create a group states *G_i _*for each entry in *f*, corresponding to the states in *M *labelled *f_i_*. A path compatible with the footprint must first go from *x*_0 _to one of the states in *G*_1_, then eventually go to *G*_2_, and on to *G_k_*. Transitions are only allowed from *G_i _*to *G_i _*or *G*_*i*+1_.

Let *f *= *f*_1_, . . . , *f_k _*be the footprint of *λ*. We create *k *groups of states *G*_1_, . . . , *G_k_*, each corresponding to the label set , of states with label *f_i_*, for each position in the footprint. Each state in  is represented in *G_i_*. States in the new model have exactly the same emission probabilities as in *M*, but we may only make transitions from states in *G_i _*to those in *G_i _*or *G*_*i*+1_, with the same transition probabilities as in *M*. (To make a proper HMM, we can create a "dump" state for paths in *M *that do not respect the footprint we seek, or not bother.) See Figure 4.

We also restrict the set of possible states visited at each position of the sequence. We require that transition from the initial state has to be made to one of the states in *G*_1_. The other entries in the first column of the forward table will be set to 0. Likewise, states from group *G_i _*can only be visited at positions *b*_*i*-1 _- *r *to *b_i _*+ *r *-1 - to ensure that the shift from *G_i _*to *G*_*i*+1 _occurs between *b_i _*- *r *and *b_i _*+ *r*. Finally, we require that the state at the last position of the sequence is in group *G_k_*. The standard forward algorithm computes the probability of *B*(*λ*, *r*) on this HMM.

Let *L *be the size of the largest label set. The runtime of this algorithm is where *t_i _*is the number of allowed labels at the *i*-th position of the sequence. For *r *= 0, which corresponds to computing the probability of a single labelling, we only allow 1 label at each position, so computing the probability of a labelling requires *O*(*L*^2^*n*) time. Now observe that increasing *r *by 1 increases the overall number of active labels by at most 2*k*. This gives us the runtime of *O*(*L*^2^(*n *+ *kr*)). In the case of jpHMMs for HIV recombination detection, this runtime is small compared to the standard forward algorithm because L ≈ *m */14 (there are 14 subtypes and sub-subtypes represented in the jpHMM for HIV) and both *k *and the chosen value of *r *are small compared to *n*.

### Optimization

Finding the most probable ball of radius *r *is NP-hard even when we restrict the centres to being consistent with a fixed footprint [9]. In practice, however, we can find high-probability balls efficiently by sampling from the distribution of state paths and using local search. If there exists a high-probability ball in the space of paths, we likely sample a path within that ball; then, the local search procedure moves us towards the centre of the ball. We used this method to predict transmembrane protein topology [9]. Unfortunately, our previous local search algorithm requires performing many forward and backward passes, which makes it too computationally expensive for use with jpHMMs. Here, we present a more efficient local search algorithm that exploits the fact that the distances between breakpoints are large.

If the size of the footprint is 2 (only one breakpoint), we can recover the most probable ball of radius *r *by doing exhaustive search. We could calculate the ball probability for every possible placement of the breakpoint, for every choice of two recombining subtypes. This requires (*n *- 1|Λ|^2 ^ball computations, or a total runtime of *O*(*n*^2^*L*^2^|Λ|^2^), which is too large.

We now reduce this runtime. For any HMM *M*, let  be the probability that *M*, started at the initial state *x*_0_, emits *y*_1_, . . . , *y_i _*while visiting only states with label *h *and that *x_i _*= *k*. Obviously, *ϕ_i,h_*(*k*) = 0 if the label of state *k *is not *h*. Analogously, let  be the probability that *M*, started at state *k*, emits *y*_*i*+1 _. . . *y_n _*while visiting only states labelled with *j*. For a labelling *λ*(*h*, *j*, *i *+ 1) consisting of two intervals labelled *h *and *j *with a breakpoint at position *i *+ 1, the probability of *λ*(*h*, *j*, *i *+ 1) is the dot product of *ϕ*_*i*,*h *_and *β*_*i*,*j*_. We obtain all forward vectors *ϕ*_*i*,*h *_by running the algorithm for computing the probability of a labelling for a labelling where all positions are labelled with *h*. Similarly, we obtain the backward vectors *β*_*i*,*j *_by running a similar backward algorithm for a labelling consisting only of *j*. Once we have all the vectors, we can find the most probable ball by a linear scan. The overall runtime of this procedure amounts to *O*(*nL*^2^|Λ|^2^).

In many cases, this runtime can be further improved if we know the location of some reference ball *B*(λ', *r*) with posterior probability over 0.5. Since the path probabilities add up to 1, we know that the maximum probability ball must intersect *B*(λ', *r*) The centres of intersecting balls must have distance at most 2*r*, and the footprints must be the same, so we are left with only 4*r *+ 1 candidate ball centres as opposed to *n *-1. We need to compute only around 6*r *dot products, giving runtime *O*(*nL*^2^). The asymptotic runtime is dominated by the forward and backward passes and not dependent on *r *since computing each dot product takes *O*(*L*) time, so computing dot products at all positions would take *O*(*nL*) time. Nevertheless, considering only 6*r *positions yields a substantial practical improvement in the constant factor.

The initial guess about the location of the reference ball can be obtained by sampling several paths from the conditional distribution  using standard algorithms [25,26] and computing the probability of a ball containing all (or most) of the sampled paths.

Similar exhaustive search procedures quickly become impractical as the number of breakpoints grows. However, we can use this approach as a heuristic to optimize each breakpoint separately. If distances between subsequent breakpoints are large, compared to the ball radius, there may be very little dependence between subsequent breakpoints, so optimizing for each breakpoint separately yields a good approximation to the globally optimal ball centre. For the first breakpoint, computing the probability of the breakpoint occuring at every position is carried out as in the one-breakpoint case except that we assume that the borders 2 to *k *occur within a reference ball for which backward table values were previously calculated. As we move to the next breakpoint, we calculate the forward values based on the optimal location of previous breakpoints, while the backward vectors still come from the calculation for the reference ball. This way, our algorithm only requires performing one additional forward pass after the backward pass for the reference ball has been performed.

Our complete optimization algorithm is as follows. First, we sample 10 paths from the HMM to determine a reference ball for our local search. We determine the majority footprint in the sample set and discard samples with a different footprint. We estimate the mean and variance of each breakpoint position from the sample. From this estimate, we derive a biased estimate of the standard deviation which we multiply by to get a biased estimate of the ball radius *r*_0.5 _such that a ball centered at the mean labelling with radius *r*_0.5 _has 0.5 posterior probability (the factor of  follows from Chebyshev's inequality). We then compute the probability of the ball centered at the mean labelling with radius  (in case where there are multiple breakpoints, we take the maximum  across all estimates). For each breakpoint, we perform the optimization procedure described above.

### Choosing the ball radius adaptively

The choice of *r *in the algorithm above may greatly influence the results, as we shall see in our experimental results. For breakpoints which occur in places where the two subtypes are highly similar, the uncertainty about breakpoint positions will be greater than in low-similarity regions. Thus, we may automatically adapt the ball size to the amount of uncertainty in a particular breakpoint. For a sequence with many breakpoints, the uncertainty about breakpoint positions may vary between breakpoints, so we alter our definition of balls to accomodate this.

An *interval region R*(*f*, [*v*_1_, *w*_1_], . . . , [*v_k_*, *w_k_*]) for a footprint *f *= *f*_1_, . . . , *f_k _*and a set of intervals [*v*_1_, *w*_1_], . . . , [*v_k_*, *w_k_*] is a set of labellings defined as follows:

For each breakpoint, we require that the interval chosen for that breakpoint has overall probability of at least half of the probability if that breakpoint was free to occur anywhere in the sequence. In other words, if we start from the reference ball as our initial interval region, then the probability is decreased at most by half each time we update an interval. Subject to that requirement, we identify the smallest possible interval for each breakpoint. This enables intervals for different breakpoints to have different width, thus accomodating different levels of uncertainty about the position of different breakpoints.

The algorithm for finding small interval regions is analogous to the algorithm for finding most probable balls. As in the previous case, we optimize each interval separately after computing the probability of the breakpoint occuring at each position of interest. We find the optimal interval for a single breakpoint with a simple greedy linear-time algorithm: traverse the list of probabilities {*p*_1_, *p*_2_, ..., *p_n_*} and maintain a variable *v*. At step *i*, *v *denotes the maximal index such that  (if no such index exists, we set *v *= 0). When the algorithm moves to the (*i *+ 1)-st step, *v *is increased if possible. The minimum interval corresponds to the minimum value of *i *- *v *found during the execution of the algorithm.

### Reducing the number of active states by beam search

Since our jpHMM has over 300, 000 states, running the standard forward and backward algorithms is computationally infeasible. We speed up the runtime of the algorithm by noticing that, at any sequence position, most entries in the forward table contain values very close to zero. Ignoring those entries has little impact on predictions but can greatly reduce computation time. *Beam search *[21] limits the number of active states at each position, by ignoring every entry in the dynamic programming table whose value is below ξp* where p* is the entry with the highest value and *ξ *is some predefined threshold. Following the decision made by the creators of jpHMM [7], we set *ξ *to 10^-20^.

## Results and Discussion

### Data used

We have evaluated the performance of our local search algorithms on several semi-synthetic data sets and one real data set. All of the semi-synthetic data sets were produced by artificially recombining real viral sequences from different subtypes. We have also run our algorithms on real recombinants and compared their predictions to published breakpoints and to the predictions made by other algorithms. It is not possible to fully evaluate our approach on real recombinants because their precise breakpoints are unknown, and human annotations are performed with the assistance of a variety of tools including ones we are comparing our work against [27]. By artificially recombining real sequences, we hope to create data sets that resemble real sequences as closely as possible while still having known ground truth values. Our implementations are available at http://www.cs.uwaterloo.ca/~jmtruszk/jphmm_balls.tar.gz.

We compared our method to the standard implementation of jpHMM decoding using the Viterbi algorithm, and to DualBrothers [13], which was chosen as a representative of phylogeny-based methods for recombination detection. We also tried to run ST-HMM [20], but it appears prohibitively slow for this task. DualBrothers takes as input a multiple alignment of subtype reference sequences and the query sequence and outputs a collection of trees together with their posterior probabilities for each column of the alignment. We chose one sequence from each subtype to serve as reference. For each query sequence, we used MAFFT [28] to align it to the reference sequences. We then ran the MCMC sampler for 50000 steps, discarding the initial 25000 steps as burn-in. For each position in the query sequence, its subtype was assigned by taking the most probable phylogeny at this position and finding the subtype that was topologically closest to the query sequence in that phylogeny. We broke ties arbitrarily.

We used three data sets from Schultz *et al*. [8] which we call the 1000-1000, 500-1500 and 300-1500 data sets. In all data sets, each artificial sequence was created by taking two real sequences from different subtypes, aligning them and composing them into one sequence by alternating intervals from each parent sequence. For example, in the 300-1500 data set, the first interval consisted of roughly 300 initial positions from the first sequence, followed an interval of length 1500 from the second sequence starting immediately after the base that was aligned to the last base from the first interval. The third interval had 300 positions taken from the first sequence and so on. The parental sequences were taken from the 6 most common HIV subtypes (A, B, C, D, F, G) and CRF 01_AE. Each of these three data sets has 40 sequences generated this way.

In another data set from Schultz [29], with single breakpoints, each sequence consisted of two intervals taken from two parental sequences. The breakpoint locations were chosen uniformly at random. Here, parental sequences from all subtypes and sub-subtypes were used. This data set contains 106 sequences. In a second set of experiments, we tested the ability of the Viterbi algorithm and our ball algorithms to identify short inserted regions in the sequence that come from a different subtype than the rest of the sequence. If a region is too short, it will not be detected by any algorithm since the model will favour paths that do not make two low-probability jumps into and out of a different subtype. Our ball algorithms should be more capable of identifying such short regions. To test this hypothesis, we generated sequences with two breakpoints, with a fixed distance between the breakpoints. Each sequence was a recombinant of two out of eleven most common subtypes, with one sequence for every choice of the two subtypes. We generated four data sets this way for different insertion lengths (see Additional file 1).

In the final set of experiments, we run all the algorithms on 20 real CRFs taken from the Los Alamos HIV sequence database [27]. We used the reference strains for CRFs 2 to 9, 11 to 21, and CRF 23. When comparing the predictions to the published breakpoints, we discarded the 5 CRFs whose annotation had been produced with the aid of jpHMM for a more fair comparison; we also discarded all CRFs whose annotation contained intervals labelled as ambiguous or unknown. This produced a set of 9 CRFs. When comparing the predictions of various algorithms, we used the whole data set of 20 CRFs.

We used two accuracy measures to evaluate the algorithms. For each true breakpoint recovered by an algorithm, we calculated the distance between the predicted and actual (or reported) breakpoint positions. We report the median of these distances for each experiment. In addition, we report the breakpoint sensitivity, defined as the fraction of the true breakpoints that were identified by an algorithm. We consider a true breakpoint identified if a prediction contains a breakpoint within 300 positions of the true breakpoint and the subtypes on both sides of the predicted breakpoint match the true subtypes. If the prediction contains a breakpoint that does not correspond to a true breakpoint in the above way, we report it as a false positive.

### Overall results

Table 1 shows the median breakpoint error for the first set of experiments. For *r *= 5 and *r *= 7, our algorithms outperformed Viterbi predictions in terms of the median distance between predicted and actual breakpoints. The adaptive region algorithm gave similar results, but it did not offer any further improvement over the ball algorithms for the above parameter choices. DualBrothers had much higher median distance than all algorithms based on the jpHMM.

**Table 1:** Comparison of different algorithms

Method	Single breakpoint	1000-1000	500-1500	300-1500
Viterbi	7 (3..17)	6 (2..17)	7 (2..20)	7 (3..16)
max ball, *r *= 1	6 (2..16)	6 (2..18)	7 (1..19)	7 (2..16)
max ball, *r *= 2	5 (2..16)	6 (1..17)	6 (2..18)	6 (2..15)
max ball, *r *= 5	**4 (1..12)**	**5 (3..14)**	**5 (3..15)**	**5 (2..13)**
max ball, *r *= 7	**4 (2..12)**	**5 (3..14)**	6 (3..14)	6 (3..12)
max ball, *r *= 10	6 (3..10)	7 (3..14)	8 (3..14)	7 (3..10)
max ball, *r *= 15	7 (3..11)	9 (4..14)	10 (5..15)	7 (4..12)
min region	**4 (1..13)**	**5 (2..14)**	6 (2..16)	**5 (2..14)**
DualBrothers	57(45..71)	51 (42..67)	48(37..54)	45 (40..51)

Shown are the median and interquartile range of differences between predicted and actual breakpoints for the four initial experiments.

We used the Wilcoxon signed rank test to compare the distribution of breakpoint errors between Viterbi and ball algorithms. For *r *= 5, the *p*-values for the four data sets in Table 1 are 4 ·10^-10^, 3 ·10^-8^, 3 ·10^-6^, and 3 ·10^-14^, respectively.

The HMM algorithms all managed to reconstruct the vast majority of breakpoints, while DualBrothers did not. Our ball algorithms noticeably outperformed Viterbi on the 300-1500 data set. For the other data sets, the differences were negligible. Table 2 shows the sensitivities for all experiments. In this set of experiments, no false positives were found for Viterbi. Our algorithms produced no false positives on all data sets except the 500-1500 data set, where each ball algorithm produced 2 false-positive breakpoints. DualBrothers recovered less than half of the breakpoints on each data set except the first and also gave a considerable number of false positives (120,122,130 and 104 for single breakpoint, 1000-1000, 500-1500 and 300-1500 data sets, respectively). DualBrothers tended to make the most mistakes on sequences derived from subtype A, often confusing it with CRF_01.

**Table 2:** Number of breakpoints recovered

Method	Single breakpoint	1000-1000	500-1500	300-1500
Viterbi	105/106	359/360	327/360	344/360
max ball, *r *= 5	105/106	360/360	326/360	350/360
max ball, *r *= 7	105/106	360/360	326/360	350/360
max ball, *r *= 10	105/106	360/360	326/360	350/360
max ball, *r *= 15	105/106	360/360	326/360	350/360
min region	105/106	360/360	326/360	350/360
DualBrothers	76/106	146/360	81/360	27/360

Shown are the fractions of recovered breakpoints for each experiment.

In the second set of experiments, we see that the accuracy of both Viterbi and ball algorithms deteriorates gradually as interval length decreases. Ball algorithms managed to identify more correct breakpoints for all interval lengths. For all algorithms, the number of false-positive breakpoints increases as intervals get smaller, with ball algorithms yielding slightly higher false-positive rates than Viterbi. The results are shown in Table 3 and Table 4. Since the choice of ball radius does not affect the number of correctly identified breakpoints, we only report results for *r *= 5. Among the correctly predicted breakpoints, the median error rates for ball algorithms were smaller than for Viterbi similarly to the first set of experiments(results not shown).

**Table 3:** True positives on short intervals

Method	250	200	150	100
Viterbi	96/110	79/110	45/110	25/110
max ball, *r *= 5	100/110	85/110	58/110	29/110

Shown are the fractions of recovered breakpoints for different interval lengths.

**Table 4:** False positives on short intervals

Method	250	200	150	100
Viterbi	2/110	4/110	8/110	10/110
max ball, *r *= 5	2/110	4/110	11/110	14/110

Shown are the fraction of false positive breakpoints for different interval lengths.

**Figure 5:**
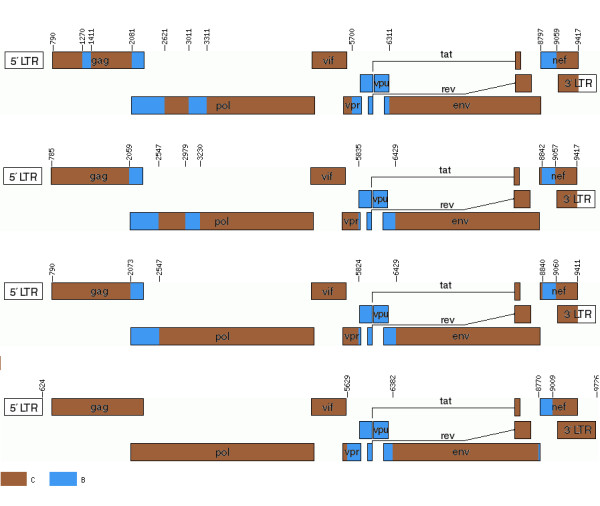
**Predicted breakpoints for CRF07_BC**. The published recombinant structure is shown on the top picture, followed by predictions from the ball algorithm, Viterbi, and DualBrothers. None of the programs recovered all of the published breakpoints. The balls algorithm did not include 2 of the published breakpoints. Viterbi missed 4 published breakpoints, whereas DualBrothers missed 6. Recombinants were drawn using the HIV recombinant drawing tool [30].

For the 9 real recombinants whose published breakpoints had not been inferred using jpHMM, the ball algorithm and Viterbi generally gave very similar results (see Table 5). Both algorithms predicted roughly half of the published breakpoints; the balls algorithm predicted two more than did Viterbi. Somewhat surprisingly, Viterbi had slightly lower median breakpoint distance to the published breakpoints, though this result is not significant according to the Wilcoxon signed-rank test (*p *= 0.42). DualBrothers recovered fewer of the published breakpoints and had substantially higher median breakpoint distance to the published breakpoints that it recovered than the other two algorithms. Figure 5 shows predictions of all the algorithms for one of the published CRFs.

**Table 5:** Comparison with published breakpoints

Method	predicted	not predicted	extra breakpoints	distance
Viterbi	39	33	5	17(4..54)
max ball, *r *= 5	41	31	5	21(6..53)
DualBrothers	22	50	19	65(37..82)

For each algorithm, we report the number of published breakpoints that have been predicted by the algorithm and the number of predicted breakpoints that do not correspond to any of the published breakpoints, as well as the median distance between published and predicted breakpoints.

There was not much agreement between DualBrothers and the other two programs on all real recombinants. Only 38 out of 150 breakpoints identified by DualBrothers for all 20 recombinants were also identified by jpHMM. In contrast, 136 out of 146 breakpoints identified by the ball algorithm were identified by jpHMM. The median distance between corresponding breakpoints was 61 between DualBrothers and jpHMM, compared to 4 between balls and jpHMM. This is not surprising since the ball algorithm uses the same model parameters as jpHMM.

The balls found by our ball algorithm often contain most of the probability mass given the model and the sequence. Out of the 106 sequences in the single-breakpoint dataset, 81 of the balls found by our algorithm had over 0.5 posterior probability, for *r *= 5.

Our prototype C++ implementation of ball algorithms processed the 1000-1000 dataset in about 15.5 hours on a standard desktop computer. The approximate time to analyze a sequence (average length 9256) is 23 minutes with our ball algorithms, which compares well with the 17.5 minutes required to use the simpler Viterbi algorithm, in our prototype. Schultz *et al*. [7] achieve a substantial speedup, to 6.7 minutes per sequence, using their implementation of Viterbi, but we expect that optimizing our methods in this domain will require only small overhead, compared to their jpHMM-HIV package. The running time of DualBrothers was about 120 minutes per sequence. While a faster implementation of DualBrothers exists [14], we were not able to compile it on our system.

### Error analysis

The strikingly lower sensitivity in all experiments on the 500-1500 data set is due to an artifact of the data set rather than flaws in the algorithms. In 33 sequences from that data set, some of the true breakpoints were located after the last consensus column of the multiple sequence alignment of HIV sequences. The bases in that region are modelled by an additional insert state emitting letters according to a background frequency, so the model cannot distinguish between any two subtypes in that region. Consequently, no algorithm that uses jpHMM will be able to predict any recombination breakpoints in the unconserved region at the end of the sequence.

The higher median breakpoint error for ball algorithms with larger radii is because in many cases, most of the probability mass is concentrated in a small fragment of the most probable ball which is located away from the centre. Moving away from the concentration region might increase the ball probability by a very small amount, which causes the algorithm to pick a ball whose centre is relatively far from the region where the breakpoint is likely. On the other hand, if the ball radius is too small (e.g. *r *= 2) the algorithm often picks small regions of high probability and ignores many other likely breakpoint locations, which causes errors similar to those made by Viterbi.

## Conclusions

We have presented two novel algorithms for predicting recombinations in viral genomes. The ball algorithm identifies recombination breakpoints up to a user-defined accuracy, while the interval region algorithm attempts to choose the accuracy adaptively based on the observed uncertainty of breakpoint locations. Both algorithms account for the inherent uncertainty about precise breakpoint locations. This leads to an improvement over the Viterbi algorithm for jpHMM in both identifying the correct breakpoints and their approximate locations. Our algorithms also show improvement on difficult instances of the problem where distances between breakpoints are small.

Both algorithms have similar accuracies in our experiments. Contrary to our expectations, the interval region algorithm does not perform better than the ball algorithm with suitable parameter choices. The interval region algorithm may still be preferred in situations where there is a lot of uncertainty about some breakpoint positions but little uncertainty about others.

## Authors' contributions

Both authors jointly developed this decoding concept. JT developed the algorithms, implemented them all, performed the experimental work and wrote most of the manuscript. DB directed the project and wrote some of the manuscript. Both authors read and approved the final manuscript.

## Supplementary Material

Additional file 1 **Data sets**. This file contains sequences and their annotations for the single-breakpoint and short interval data sets. Click here for file
